# MRTO4 acts as an independent prognostic and immunological biomarker and is correlated with tumor microenvironment in hepatocellular carcinoma

**DOI:** 10.1590/1414-431X2024e13780

**Published:** 2024-11-04

**Authors:** Baobao Liang, Lan Li, Chenyang He, Meng Wang, Guochao Mao

**Affiliations:** 1 Department of Oncology, The Second Affiliated Hospital of Xi'an Jiaotong University, Xi'an, Shaanxi, China; 2 Department of Breast Surgery, Shaanxi Provincial Cancer Hospital, Xi'an, Shaanxi, China

**Keywords:** MRTO4, Clinicopathological features, Immune cell infiltration, Immunophenoscore, Tumor microenvironment, Hepatocellular carcinoma

## Abstract

Liver cancer is a malignant tumor found worldwide. mRNA turnover 4 homolog (*MRTO4*) is highly expressed in hepatocellular carcinoma (HCC) tissues, and we explored its relationship with HCC. All cancer data were downloaded from the Cancer Genome Atlas (TCGA), the Cancer Immune Atlas (TCIA), and the Human Protein Atlas (THPA). Stromal scores, immune scores, and ESTIMATE scores were calculated by “ESTIMATE” R package. Single sample gene set enrichment analysis and CIBERSORT were used to evaluate the immune status and infiltration of cancer tissues. pRRophetic R package was used to predict the half-maximal inhibitory concentration (IC_50_) of different drugs in each sample. *MRTO4* overexpression was associated with poor prognosis in HCC, and positively correlated with the stage and grade of HCC patients. The average immunophenoscore (IPS) of the low *MRTO4* group was significantly higher than that of the high *MRTO4* group. Tumor microenvironment (TME) scores were significantly higher in the low *MRTO4* group than in the high *MRTO4* group in HCC. *MRTO4* expression was positively correlated with tumor mutation burden (TMB) and was positively correlated with most immune checkpoint gene expressions in HCC. Drug sensitivity analysis showed significantly higher IC_50_ values for 5-fluorouracil, gemcitabine, and sorafenib in patients with low *MRTO4* expression than in those with high *MRTO4* expression. *MRTO4* acts as an independent prognostic and immunological biomarker and is correlated with clinical stage, tumor grade, and drug sensitivity in HCC. It may serve as a putative therapeutic target and potential biomarker for prognosis of HCC.

## Introduction

Liver cancer is the seventh most common malignant tumor globally, with about 841,000 new cases and 7.82 million deaths annually worldwide. Hepatocellular carcinoma (HCC) comprises nearly 90% of all cases of primary liver cancer (1). China is the leading country for liver cancer, with the fourth and second highest incidence and mortality rates. The causes of this poor prognosis include severe underlying liver disease, delayed diagnosis, and lack of effective treatment. Furthermore, mortality from HCC is increasing faster than any other cancer. Despite some encouraging progress, treatment options for patients with advanced HCC remain very limited. This unsatisfactory clinical management of HCC patients is largely attributed to the lack of ideal prognostic markers and therapeutic targets.

Ribosome biogenesis, as a major consumer of cellular energy, plays a key role in cellular adaptation to environmental changes and has a powerful impact on cellular metabolism and cell cycle regulation (2-4). Its regulation is associated with post-translational modifications of TAF (trans-acting factor). As a member of the TAF family, *MRTO4* is involved in eukaryotic ribosome biogenesis and is closely associated with tumors (5,6). It has been reported to be significantly overexpressed in ovarian cancer cells with aggressive biological behavior and in invasive gastric cancer (7,8). In addition, *MRTO4* was identified as one of the hub susceptibility genes for COVID-19 in lung adenocarcinoma (9) and has been shown to display significant mRNA alterations in buccal mucosa (6). However, data on the impact of *MRTO4* in HCC are lacking.

In our study, we first conducted expression analysis of *MRTO4* and assessed its relationship with clinicopathological features and prognosis. Secondly, we investigated the relationship between *MRTO4* expression and immune cell infiltration, IPS (immunophenoscore), TME (tumor microenvironment) score, TMB (tumor mutation burden), immune checkpoint gene expression, and drug sensitivity in HCC. Finally, we explored the possible biological functions and related signaling pathways of *MRTO4* and related genes in HCC. By these means, we sought to find a new gene expression marker and pathways and to reveal the essential mechanisms associated with HCC carcinogenesis.

## Material and Methods

### Data acquisition and expression analysis

All data available for analysis were acquired from the Cancer Genome Atlas (TCGA) in fragment per kilobase million (FPKM) format and then converted to transcripts per million (TPM) format. Then, we analyzed *MRTO4* expression differences in unpaired samples of all cancer species using Mann-Whitney U-test and in paired samples of liver cancer using paired sample *t*-test, and finally all results were visualized using the “ggplot2” (v. 3.3.3) R package. The confirmation of MRTO4 protein expression levels in HCC tissues is based on the Human Protein Atlas (THPA) (10), which is an antibody-based proteomic data bank of normal and cancerous tissues as a pathological tool.

### Comparison of the characteristics between the high and low *MRTO4* expression groups

The grouping method used in all analyses was to divide the samples into two groups, low *MRTO4* group and high *MRTO4* group, using the median *MRTO4* expression as the boundary. Clinicopathological characteristics including age, gender, grade, stage, T, M, and N were analyzed to determine differences between the two groups. The TNM system is the most commonly used cancer staging system, which categorizes tumors based on their size and extent (T), the involvement of nearby lymph nodes (N), and the presence of metastasis (M). The stromal scores, immune scores, and ESTIMATE scores were calculated by “ESTIMATE” R package. Additionally, CIBERSORT was used to calculate absolute immune infiltration fraction score for all primary tumor samples to reveal 22 types of immune cells in HCC (11). The GSVA package was used for ssGSEA analysis to calculate the enrichment fraction of additional immune cells between the two groups (12,13). pRRophetic is an R package that utilizes tumor gene expression levels to make predictions about clinical chemotherapy responses (14). It specifically focuses on determining the half-maximal inhibitory concentration (IC_50_) of compounds, which are obtained from the Genomics of Drug Sensitivity in Cancer (GDSC) website. By using the pRRophetic package in R software, we were able to calculate the sensitivity score of each compound for both the high-risk group and the low-risk group of patients.

### IPS analysis

The IPS refers to the four main components that determine immunogenicity (effector cells, immunosuppressive cells, MHC molecules, and immunomodulators). IPS is calculated according to gene expression in representative cell types (range 0 to 10). IPS results for TCGA-LIHC patients can be downloaded from TCIA (https://tcia.at/home).

### GO and KEGG

To analyze the biological changes of *MRTO4* expression-related genes, enrichment analysis of markers, biological processes, and the KEGG pathway was performed. Gene Ontology (GO) database was created to define and describe genes and proteins in terms of their associated molecular function, biological process, and cellular component (15,16), and KEGG is an online database for systematic analysis of gene function from the perspective of gene and molecular networks. The “clusterProfiler” R package (17) (v. 3.14.3) and the “ggplot2” R package (v. 3.3.3) were used for enrichment analysis and visualization of MRTO4-related genes, respectively. The threshold condition was set to P.adj <0.05 and *q* value <0.2.

### Statistical analysis

In our study, continuous variables were compared using the Student's *t*-test and categorical variables were compared using the Mann-Whitney U-test. Prognostic analysis of *MRTO4* was assessed by univariate and multivariate analysis, and Kaplan-Meier curves were plotted to assess the impact of *MROT4* on the survival prognosis of patients in the high and low subgroups. The log rank test was used to evaluate the difference in survival between the high expression and low expression patients. Tumor mutational burden (TMB) is a diagnostic biomarker that categorizes cancer patients' response to immune checkpoint inhibitor therapies. It represents the average number of somatic mutations per megabase in the tumor exome, correlation of MRTO4 expression with TMB, and immune checkpoint gene expression in HCC using Spearman's r value and visualization of the results using the “ggplot2” (version 3.3.3) R package. Heat maps, forest plots, and circle plots were generated using the R package “ggplot2”. P<0.05 was set as statistically significant.

## Results

### Pan-cancer analysis of *MRTO4* expression

**Figure 1:**
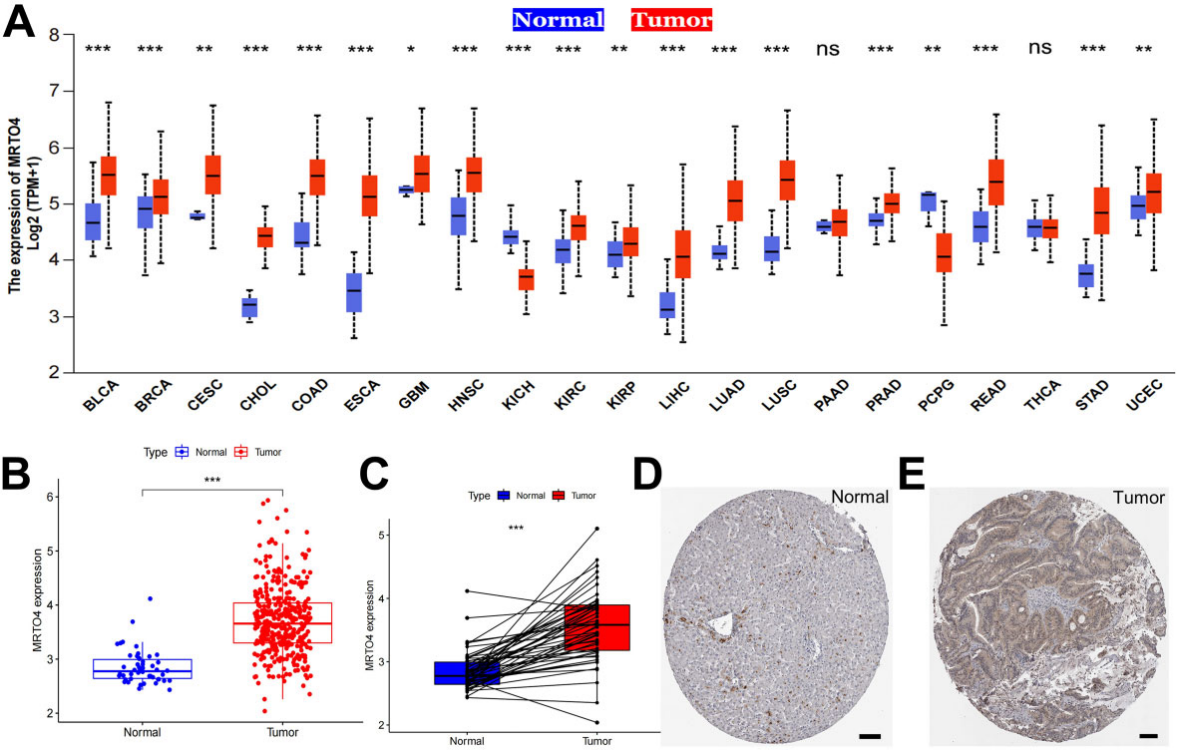
*MRTO4* expression analysis in human tumors. **A**, Pan‐cancer analysis of *MRTO4* expression in the Cancer Genome Atlas. **B** and **C**, Expression of *MRTO4* in unpaired and paired hepatocellular carcinoma (HCC) samples and normal samples. Data are reported as median and IQR. *P<0.05, **P<0.01, ***P<0.001; Mann‐Whitney U‐test. ns: no significance. **D** and **E**, MRTO4 expression in HCC and normal tissue by immunohistochemistry. Scale bar, 100 μm.

To explore the significance of *MRTO4* in human tumors, we selected a total of 21 tumors from the TCGA database. Results showed that *MRTO4* is differentially expressed in almost all tumors compared with normal tissues. As shown in Figure 1A, *MRTO4* was highly expressed in 17 tumors, including BLCA, BRCA, CESC, COAD, CHOL, ESCA, GBM, HNSC, KIRP, KIRC, LIHC (also known as HCC), LUAD, LUSC, PRAD, READ, STAD, and UCEC, and lowly expressed in KICH and PCPG, compared with normal tissues. Furthermore, we found no significant difference in the expression of *MRTO4* in PAAD and THCA. In addition, we generated scatter plots of *MRTO4* expression in the HCC group and the normal group, as shown in Figure 1B, which revealed a statistically significant increase in *MRTO4* expression in HCC compared with normal. Further paired sample analysis and data from THPA demonstrated a statistically significant increase in MRTO4 expression in HCC (Figure 1C-E). Our research findings provide strong evidence that *MRTO4* is significantly overexpressed in a wide range of tumors, including HCC, highlighting a close correlation between *MRTO4* and these malignancies.

### Prognostic evaluation of *MRTO4* in HCC

**Figure 2:**
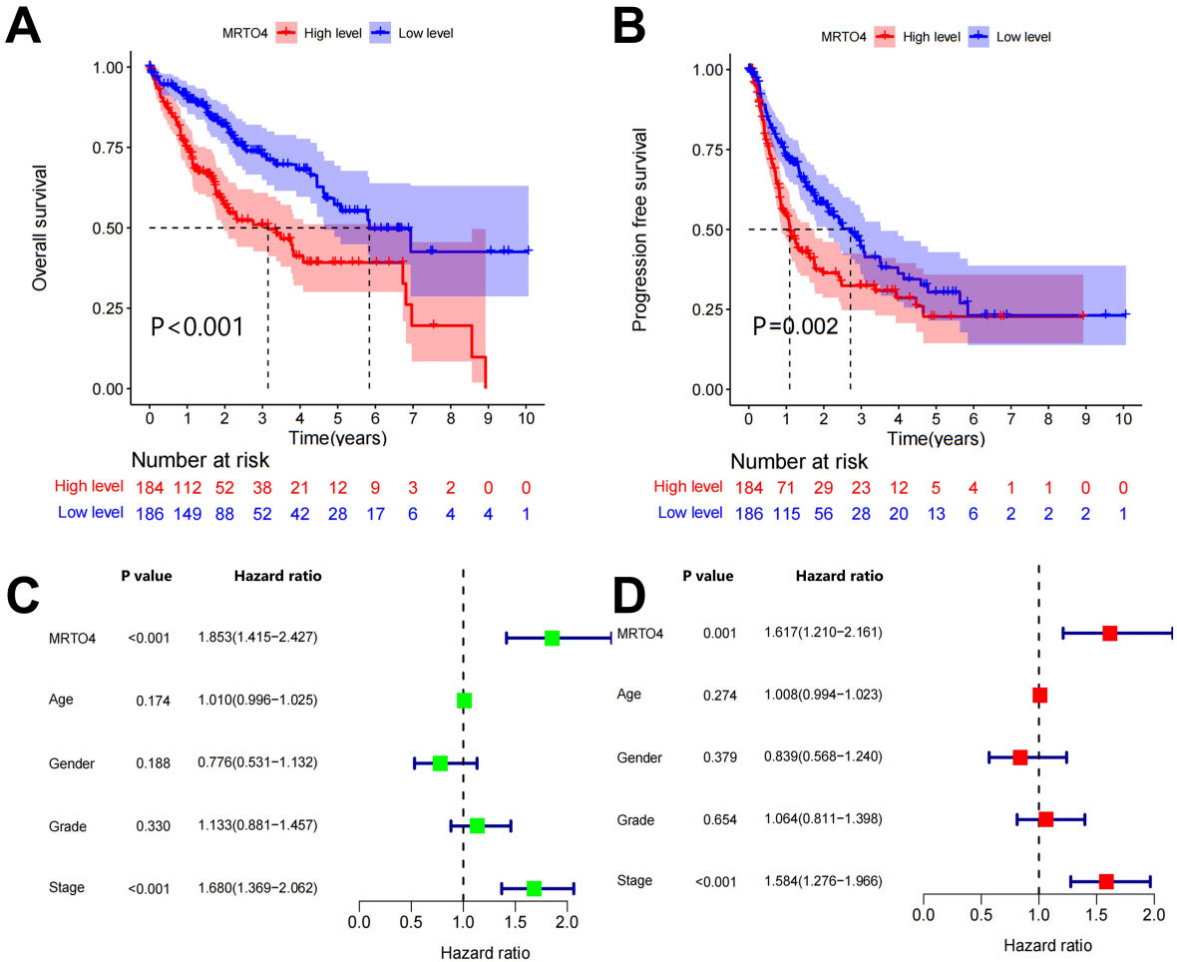
Prognostic evaluation of *MRTO4* in hepatocellular carcinoma. **A** and **B**, Kaplan‐Meier curves and log‐rank test analysis of overall survival and progression‐free survival in high‐ or low‐*MRTO4* expression groups. **C** and **D**, Univariate and multivariable analysis of *MRTO4* and various clinical factors.

To clarify the prognostic value of *MRTO4* in HCC, we evaluated the effect of *MRTO4* expression levels on progression-free survival (PFS) and overall survival (OS) in patients with HCC. As shown in Figure 2A and B, patients with HCC in the low *MRTO4* expression group had significantly better PFS (P=0.002) and OS (P<0.001) than those in the high *MRTO4* expression group. To further investigate whether *MRTO4* can independently predict the prognosis of HCC patients, we conducted univariate and multivariate analyses including *MRTO4*, gender, age, grade, and stage. As shown in Figure 2C, univariate analysis results showed that *MRTO4* and stage were negatively correlated with the prognosis of HCC patients (*MRTO4*: HR=1.853, 95%CI: 1.415-2.427, P<0.001; Stage: HR=1.680, 95%CI: 1.369-2.062, P<0.001). Subsequent multivariate analysis demonstrated *MRTO4* and stage as independent prognostic factors in patients with HCC (Figure 2D, *MRTO4*: HR=1.617, 95%CI: 1.210-2.161, P=0.001; Stage: HR=1.584, 95%CI: 1.276-1.966, P<0.001). Taken together, we demonstrated that *MRTO4* may serve as an independent prognostic biomarker for HCC patients.

### Correlation between *MRTO4* expression and clinicopathological characteristics in patients with HCC

**Figure 3:**
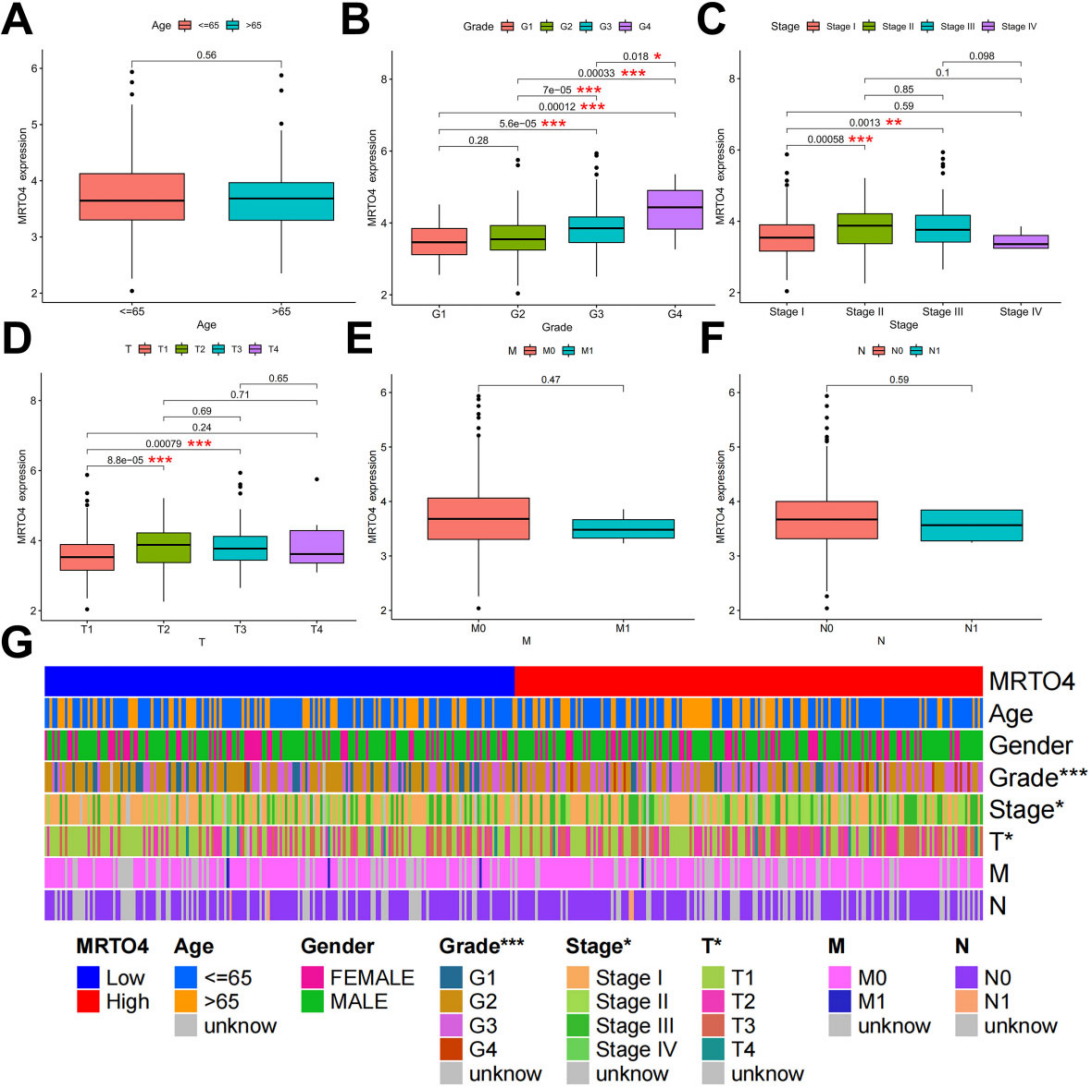
Correlation between *MRTO4* expression and clinicopathological features in hepatocellular carcinoma patients. Correlation of *MRTO4* expression with age (**A**), grade (**B**), stage (**C**), and tumor size and extent (T) (**D**), presence of metastasis (M) (**E**), and involvement of nearby lymph nodes (N) (**F**) scores. **G**, Heat map of the correlation between high‐ and low‐*MRTO4* expression groups and those features. Data are reported as median and IQR. *P<0.05, **P<0.01, ***P<0.001; Mann‐Whitney U‐test.

We used TCGA to explore the clinical significance of *MRTO4* expression in HCC patients. We found that *MRTO4* expression was statistically different in patients with HCC in terms of grade (Figure 3B), stage (Figure 3C), and T (Figure 3D). In addition, the results showed no correlation between *MRTO4* expression and age, M, and N in patients with HCC (Figure 3A, E, and F). We further analyzed whether the clinicopathological characteristics were different between high- and low-*MRTO4* groups. Heat map revealed significant differences in grade (P<0.001), stage (P<0.05), and T (P<0.05) between the two groups, while there were no differences in age, gender, M, and N (Figure 3G). Overall, our findings suggested that *MRTO4* expression was associated positively with stage and grade of HCC patients and can be used as an indicator to determine and predict the risk of HCC patients.

### Relevance of *MRTO4* expression to immune cell infiltration in HCC

**Figure 4:**
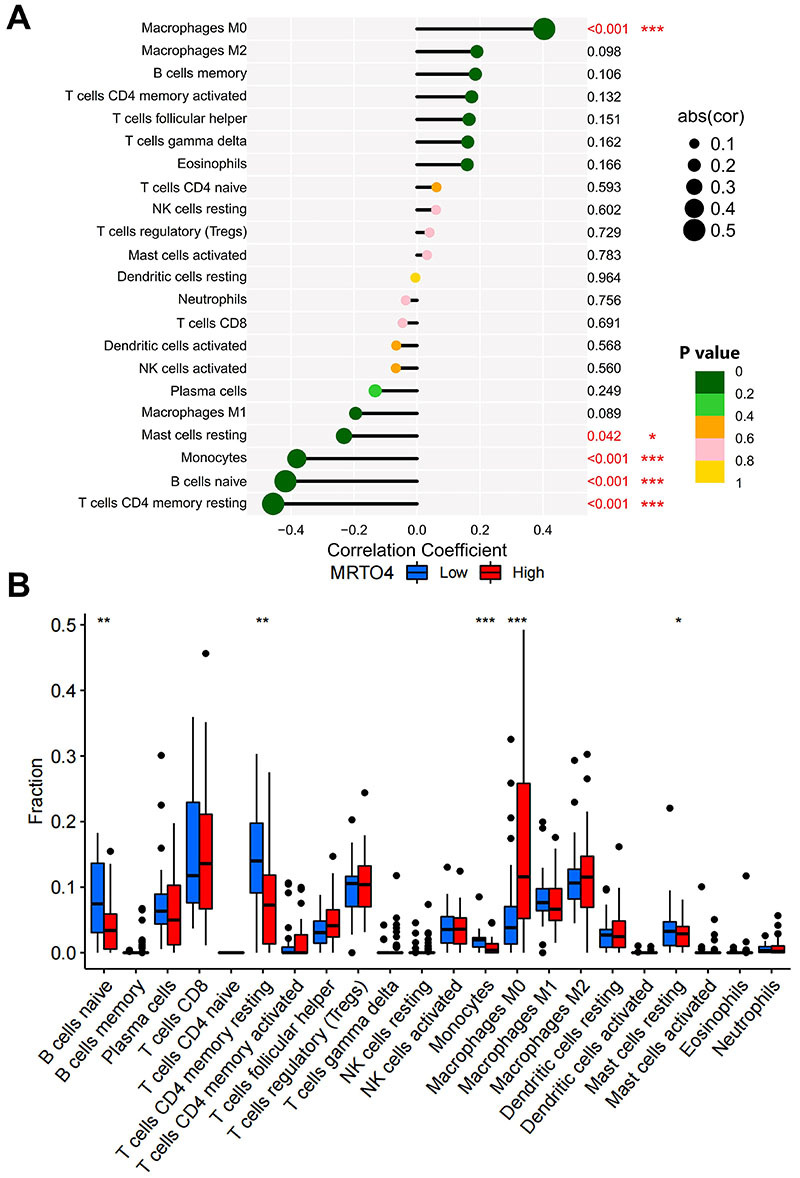
Correlation between *MRTO4* expression and immune cell infiltration in hepatocellular carcinoma. **A**, Correlation analysis of *MRTO4* expression with infiltration of various immune cells. **B**, Differences in the infiltration of various immune cells in the groups with high‐ or low‐*MRTO4* expression. Data are reported as median and IQR. *P<0.05, **P<0.01, ***P<0.001; log rank test.

To investigate whether *MRTO4* is involved in immune cell infiltration in HCC, we performed an analysis of its correlation with immune cell infiltration. We included a total of 22 immune cells, as shown in Figure 4A, and the results demonstrated that M0 macrophages (P<0.001) were the only immune infiltrating cells that positively correlated with *MRTO4* expression, whereas CD4+ memory resting T cells (P<0.001), naive B cells (P<0.001), monocytes (P<0.001), and resting mast cells (P<0.05) were negatively correlated with *MRTO4* expression. Next, we divided the samples into high and low groups according to *MRTO4* expression, and analyzed the differences in the levels of various immune cell carcinoma infiltrations between the two groups in HCC. As shown in Figure 4B, the results demonstrated that the infiltration levels of naive B cells (P<0.01), CD4+ memory resting T cells (P<0.01), monocytes (P<0.001), and resting mast cells (P<0.05) were significantly higher in the low *MRTO4* group than those in the high *MRTO4* group. In contrast, the level of M0 macrophage infiltration was significantly lower in the high *MRTO4* group (P<0.001). Taken together, our results suggested that *MRTO4* is involved in immune cell infiltration in HCC.

### Relevance of *MRTO4* expression to IPS in HCC

**Figure 5:**
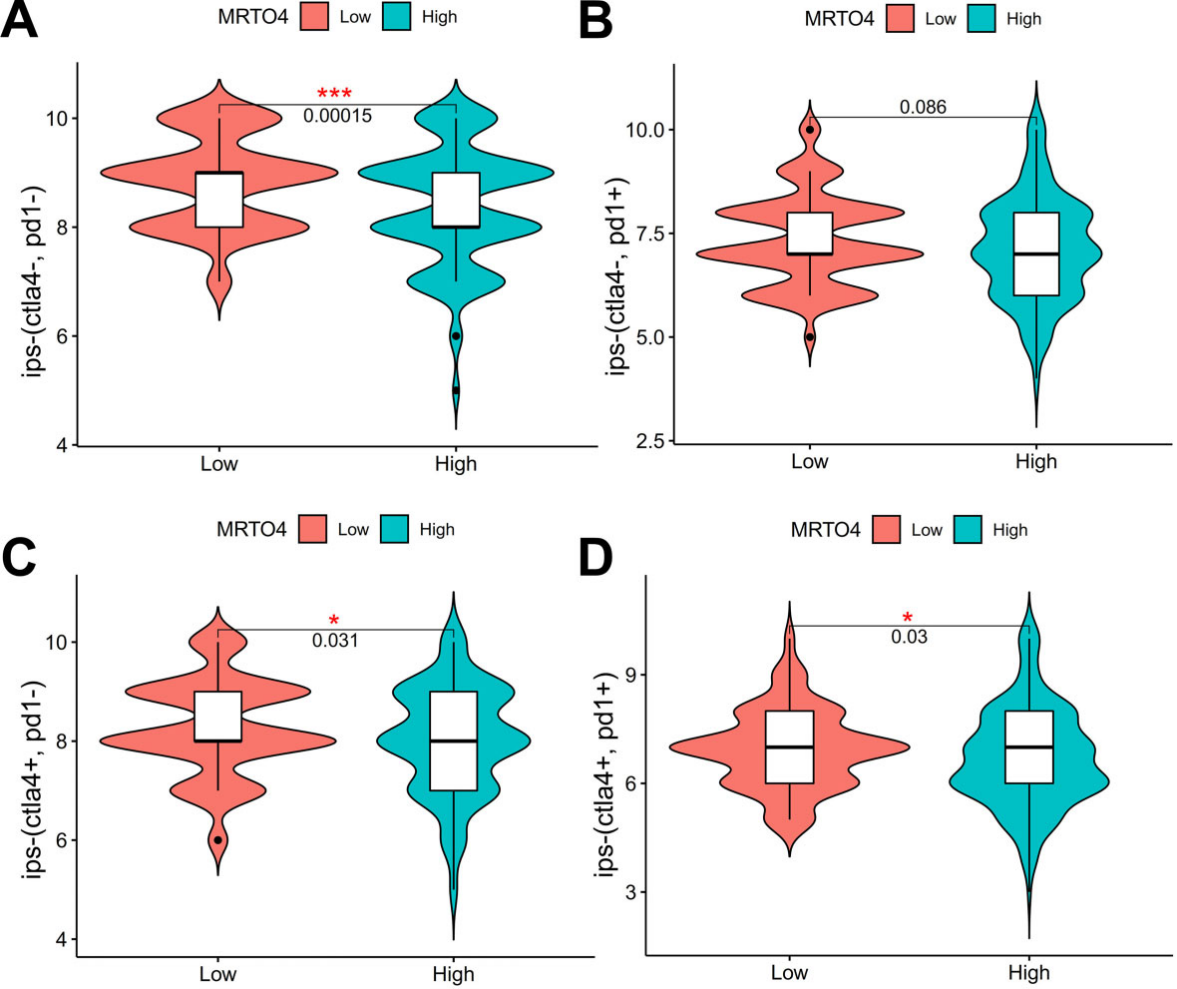
Violin plot of immunophenoscore (IPS) scores among ips‐(ctla4–, pd1–) (CTLA4 negative response and PD1 negative response) (**A**), ips‐(ctla4–, pd1+) (**B**), ips‐(ctla4+, pd1–) (**C**), and ips‐(ctla4+, pd1+) (**D**) in the respective high and low *MRTO4* groups. *P<0.05, ***P<0.001; log rank test.

To explore whether *MRTO4* expression could predict the efficacy of immunotherapy, we divided the samples into high and low groups according to *MRTO4* expression to investigate whether there was a difference in IPS between the two groups. According to the positive (+) or negative (-) response of PD1 and CTLA4, samples were divided into four groups: ips-(ctla4-, pd1-), ips-(ctla4-, pd1+), ips-(ctla4+, pd1-), and ips-(ctla4+, pd1+). Our findings showed that the average IPS of the low *MRTO4* group was significantly higher than that of the high *MRTO4* group, including ips-(ctla4-, pd1-) (P<0.001, Figure 5A), ips-(ctla4+, pd1-) (P<0.05, Figure 5C), and ips-(ctla4+, pd1+) (P<0.05, Figure 5D), while there was no significant difference in ips-(ctla4-, pd1+) (P=0.086, Figure 5B). These results suggested that *MRTO4* was valid for predicting response to PD1 and CTLA4 treatment.

### Correlation of *MRTO4* expression with TME, TMB, immune checkpoint gene expression, and drug sensitivity in HCC

**Figure 6:**
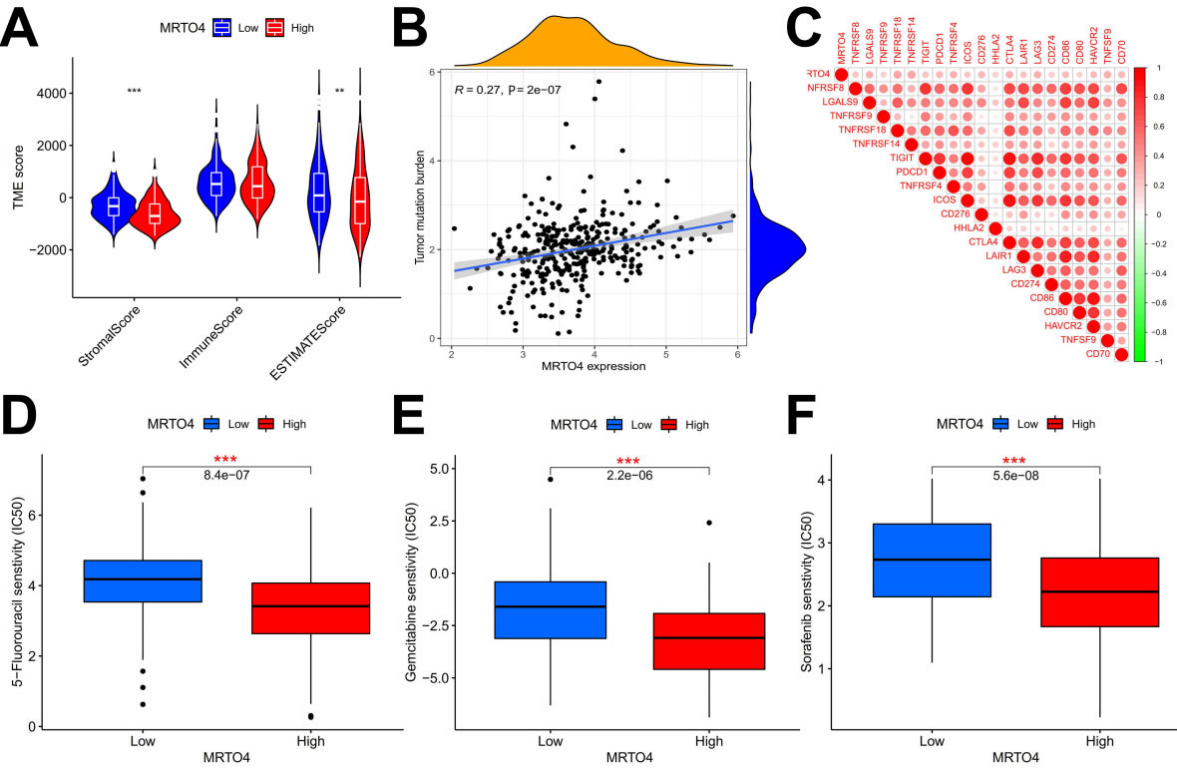
Correlation of *MRTO4* expression with tumor microenvironment (TME) score (**A**), tumor mutation burden (**B**), immune checkpoint gene expression (**C**), and drug sensitivity (**D**, **E**, and **F**) in hepatocellular carcinoma. Data are reported as median and IQR. **P<0.01; ***P<0.001; log rank test.

To investigate the possible immunological contribution of *MRTO4* in HCC, we examined the correlation between *MRTO4* expression and immunological biomarker in HCC. Our data revealed that TME scores were significantly higher in the low *MRTO4* group than in the high *MRTO4* group in HCC, and this difference was largely attributed to differences in stromal scores rather than immune scores (Figure 6A). We found that *MRTO4* expression was positively correlated with TMB (R=0.27, P=2.0e-07, Figure 6B), and TMB in malignant tumors is considered to be a novel biomarker used to predict the impact of immunotherapy on patients. In addition, the results showed that *MRTO4* was positively associated with the expression of almost all immune checkpoint genes, including *TNFRSF14*, *TNFRSF18*, *TNFSF9*, *CTLA4*, *LGALS9*, *LAG3*, *TNFRSF4*, *HAVCR2*, *CD70*, *CD276*, *CD86*, *PDCD1*, *TIGIT*, *LAIR1*, *TNFRSF8*, *HHLA2*, *CD80*, *ICOS*, *CD274*, and *TNFRSF9* (Figure 6C and Supplementary Table S1), indicating the promising use of *MRTO4* in predicting immune therapy responses. In addition, we further performed drug sensitivity analysis between the two groups. We screened a total of 3 drugs relevant to the treatment of HCC. Among them, 5-fluorouracil, gemcitabine and sorafenib had lower IC_50_ in patients with high *MRTO4* expression than in those with low expression (P=8.4e-07, Figure 6D; 2.2e-06, Figure 6E; 5.6e-08, Figure 6F; respectively), indicating that these drugs had better efficacy in the high *MRTO4* group. This suggests that *MRTO4* may provide guidance for the individualized clinical application of these drugs.

### Analysis of biological functions and signaling pathways of genes associated with *MRTO4* expression in HCC

**Figure 7:**
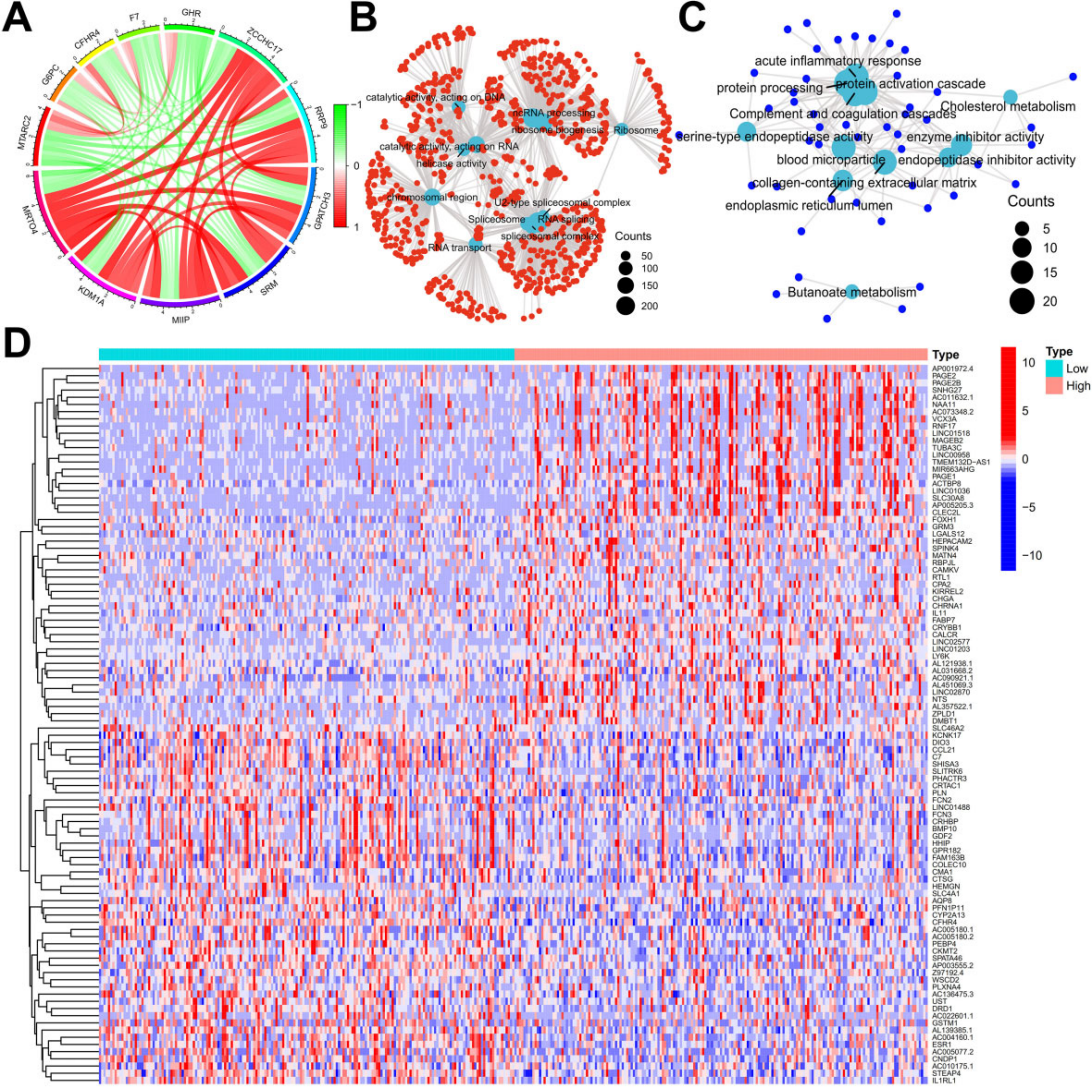
Biological functions and signaling pathways of genes associated with *MRTO4* expression in hepatocellular carcinoma. **A**, Circle plots of the top 5 genes positively and negatively correlated with *MRTO4* expression. **B** and **C**, GO and KEGG analysis of genes positively and negatively correlated with *MRTO4* expression. **D**, Heat map of the top 50 differentially expressed genes in the two groups according to *MRTO4* expression.

To investigate the biological role of *MRTO4* in HCC, we first carried out a genome-wide gene mapping correlation analysis and obtained a total of 4024 genes associated with *MRTO4* expression in TCGA, of which 3952 were positively associated and 72 were negatively associated genes. In addition, we produced a circular map of *MRTO4* expression-associated genes (Figure 7A). The top six genes positively associated with *MRTO4* expression were *KDM1A*, *MIIP*, SRM, *GPATCH3*, *RRP9*, and *ZCCHC17*, while the top five genes negatively associated with MRTO4 expression were *MTARC2*, *G6PC*, *CFHR4*, *F7*, and *GHR* (Supplementary Table S2). We further analyzed and obtained differentially expressed genes between groups based on *MRTO4* expression. The heat map in Figure 7D shows the top 50 significantly upregulated and downregulated differentially expressed genes in both groups. To further explore the biological functions and signaling pathways involved in *MRTO4* and its expression-related genes, we performed GO and KEGG analysis. Our findings showed that genes positively associated with *MRTO4* expression were mainly enriched in catalytic activity, spliceosome, RNA splicing, ncRNA processing, and ribosome, while genes negatively associated with *MRTO4* expression were mainly enriched in protein processing, protein activation cascade, enzyme inhibitor activity, and collagen-containing extracellular matrix (Figure 7B and C). Taken together, these findings suggested that *MRTO4* may contribute to the development of HCC by mediating RNA synthesis and catalytic activity, thereby participating in cancer-related signaling pathways.

## Discussion

To our knowledge, this is the first study conducted in patients with HCC to study *MRTO4* expression, prognostic value, drug sensitivity information, and its relationship with immune infiltration. Poor prognosis of HCC is attributed to the lack of ideal prognostic markers and therapeutic targets. Therefore, identifying effective prognostic biomarkers is key to improving the efficacy of targeted therapy and reducing the poor prognostic impact of HCC. We found that *MRTO4* was upregulated in patients with HCC and could serve as an independent marker for a poor prognosis. Furthermore, *MRTO4* expression was associated positively with the stage and grade of HCC patients. All these findings suggested an important role of *MRTO4* in HCC and its possible involvement in the development of HCC and as a promoter of hepatocarcinogenesis.

Various chronic inflammatory conditions, including chronic inflammation, alcoholic steatohepatitis, nonalcoholic fatty liver, and exposure to toxic substances, cause tumor-immune system dysfunction by disrupting the reticuloendothelial system of the liver (18), which in turn leads to an immunosuppressive TME and a state of host immunosuppression, resulting in tumor immune evasion. Multiple mechanisms, including upregulation of suppressor immune cells (regulatory T cells (Tregs), myeloid-derived suppressor cells (MDSCs), and tumor-associated macrophages (TAMs)), downregulation of antitumor effector cells (dendritic cells (DCs) and natural killer cells (NKs)) (19), the intricate cytokine environment, and upregulation of immune checkpoint proteins (20), interact to characterize immunosuppression in the TME. Researchers have made important advancements in liver cancer research by targeting immune cells and developing immunomodulators for cancer therapy. These immunomodulators, known as immune checkpoint inhibitors (ICIs), have shown great promise in treating liver cancer. By understanding the different roles that immune cells play in various pathways of liver cancer, researchers have been able to develop ICIs that specifically target and enhance the immune response against cancer cells. This breakthrough discovery has opened up new possibilities for effective treatment strategies in liver cancer (21). Interestingly, overexpression of immune checkpoint-associated molecules in HCC patients due to long-term chronic inflammation leads to apoptosis of CD8+ T cells and reduced anti-tumor activity of immune cells (22,23). However, ICIs can reverse this situation by blocking ligand-binding sites or inhibiting the expression of immune checkpoint-associated molecules, thereby promoting the proliferation of immune cells and enhancing anti-tumor immune responses, thus becoming promising drugs for the treatment of cancer.

Immunotherapy monotherapies currently suffer from relatively low response rates. Therefore, the combination of multiple ICIs or immunotherapies with other therapies, such as anti-angiogenic drugs (24), to obtain additional efficacy through further synergy, has emerged as a new strategy for the treatment of HCC. These include: combination with anti-angiogenic drugs, combination with local ablation therapy, dual therapy with PD-1 and CTLA-4 inhibitors, and dual therapy with checkpoint and multi-kinase inhibitors. For example, in 2020, the Food and Drug Administration in the United States approved the use of nivolumab plus ipilimumab for the treatment of patients with HCC who have previously received sorafenib (25). The combination of atezolizumab, an immune checkpoint inhibitor, and bevacizumab, an anti-angiogenic agent, is the first treatment regimen to demonstrate improved overall survival in advanced-stage HCC patients compared to sorafenib (26). Many clinical trials are underway to validate the clinical efficacy and safety of the above combination regimens, and additional phase III study results are needed to draw more conclusions. We found that *MRTO4* expression was positively correlated with M0 macrophages and negatively correlated with CD4+ memory resting T cells, resting mast cells, naive B cells, and monocytes in HCC. In the immune checkpoint screen, we found that *MRTO4* was positively associated with the expression of almost all immune checkpoint genes, including *TNFRSF14*, *TNFRSF18*, *TNFSF9*, *CTLA4*, *LGALS9*, *LAG3*, *TNFRSF 4*, *HAVCR2*, *CD70*, *CD276*, *CD86*, *PDCD1*, *TIGIT LAIR1*, *TNFRSF8*, *HHLA2*, *CD80*, *ICOS*, *CD274*, and *TNFRSF9*, indicating the potential use of *MRTO4* in predicting biomarkers of immunotherapeutic response. Together, these results suggested that *MRTO4* together with the various immune infiltrating cells in the TME influence each other and together form part of a complex HCC ecosystem. Our results are derived from database analysis and therefore necessitate further laboratory research and validation.

### Conclusion

In conclusion, we clarified that *MRTO4* is an independent biomarker for prognosis and associated with clinical stage, tumor grade, immune cell infiltration, IPS, TME score, TMB, drug sensitivity, and immune checkpoint gene expression, and we explored the biological functions and signaling pathways of *MRTO4* in HCC. However, validation of these findings on large cohorts is warranted.

## Supplementary Material

Click here to view [pdf].
