# Supplementary material for: MRTO4 acts as an independent prognostic and immunological biomarker and is correlated with tumor microenvironment in hepatocellular carcinoma
Source: Braz J Med Biol Res. 2024 Nov 4;57:e13780. doi: 10.1590/1414-431X2024e13780 (PMC11540254; doi:10.1590/1414-431X2024e13780)
Supplement: Supplementary file 1 [file 1414-431X-bjmbr-57-e13780-suppl.pdf]

Table S1. Immune checkpoint genes positively correlated with *MRTO4* expression.

Gene	Gene name	Ensemble ID	Gene type	Function
TNFRSF14	TNF Receptor Superfamily Member 14	ENSG00000157873	Protein coding	Activate inflammatory and inhibitory T-cell immune response
TNFRSF18	TNF Receptor Superfamily Member 18	ENSG00000186891	Protein coding	Increase expression upon T-cell activation
TNFSF9	TNF Superfamily Member 9	ENSG00000125657	Protein coding	Involved in the antigen presentation process and in the generation of cytotoxic T cells
CTLA4	Cytotoxic T-Lymphocyte Associated Protein 4	ENSG00000163599	Protein coding	Transmit an inhibitory signal to T cells
LGALS9	Lectin, Galactoside-Binding, Soluble, 9	ENSG00000168961	Protein coding	Implicated in modulating cell-cell and cell-matrix interactions
LAG3	Lymphocyte Activating 3	ENSG00000089692	Protein coding	Inhibitory receptor on antigen activated T-cells
TNFRSF4	TNF Receptor Superfamily Member 4	ENSG00000186827	Protein coding	Activate NF-kappaB
HAVCR2	Hepatitis A Virus Cellular Receptor 2	ENSG00000135077	Protein coding	Generally accepted to have an immunosuppressive function
CD70	CD70 Molecule	ENSG00000125726	Protein coding	Induce proliferation of costimulated T cells
CD276	CD276 Molecule	ENSG00000103855	Protein coding	Participate in the regulation of T-cell-mediated immune response
CD86	CD86 Molecule	ENSG00000114013	Protein coding	A costimulatory signal for activation of the T-cell
PDCD1	Programmed Cell Death 1	ENSG00000188389	Protein coding	An immune-inhibitory receptor expressed in activated T cells
TIGIT	T Cell Immunoreceptor With Ig And ITIM Domains	ENSG00000181847	Protein coding	Suppresses T-cell activation
LAIR1	Leukocyte Associated Immunoglobulin Like Receptor 1	ENSG00000167613	Protein coding	An inhibitory receptor found on peripheral mononuclear cells, including natural killer cells, T cells, and B cells
TNFRSF8	TNF Receptor Superfamily Member 8	ENSG00000120949	Protein coding	Regulates gene expression through activation of NF-kappa-B
HHLA2	HHLA2 Member Of B7 Family	ENSG00000114455	Protein coding	Regulate cell-mediated immunity
CD80	CD80 Molecule	ENSG00000121594	Protein coding	Induce T-cell proliferation and cytokine production
ICOS	Inducible T Cell Costimulator	ENSG00000163600	Protein coding	Cell-cell signaling, immune responses, and regulation of cell proliferation
CD274	CD274 Molecule	ENSG00000120217	Protein coding	Inhibit T-cell activation and cytokine production
TNFRSF9	TNF Receptor Superfamily Member 9	ENSG00000049249	Protein coding	Contribute to the clonal expansion, survival, and development of T cells

Table S2. Top genes positively or negatively associated with *MRTO4* expression.

Gene	Gene name	Ensemble ID	Gene type	Function
KDM1A	Lysine Demethylase 1A	ENSG00000004487	Protein coding	A component of several histone deacetylase complexes
MIIP	Migration And Invasion Inhibitory Protein	ENSG00000116691	Protein coding	An inhibitor of cell migration and invasion
SRM	Spermidine Synthase	ENSG00000116649	Protein coding	A mediator of cell growth and differentiation
GPATCH3	G-Patch Domain Containing 3	ENSG00000198746	Protein coding	Enable nucleic acid binding activity
RRP9	Ribosomal RNA Processing 9, U3 Small Nucleolar RNA Binding Protein	ENSG00000114767	Protein coding	A component of the nucleolar small nuclear ribonucleoprotein particle
ZCCHC17	Zinc Finger CCHC-Type Containing 17	ENSG00000121766	Protein coding	Involved in RNA stabilization
MTARC2	Mitochondrial Amidoxime Reducing Component 2	ENSG00000117791	Protein coding	Reduce N-hydroxylated substrates
G6PC	Glucose-6-Phosphatase Catalytic Subunit 1	ENSG00000131482	Protein coding	Transporters for G6P, inorganic phosphate, and glucose
CFHR4	Complement Factor H Related 4	ENSG00000134365	Protein coding	Involved in complement regulation
F7	Coagulation Factor VII	ENSG00000057593	Protein coding	A vitamin K-dependent factor essential for hemostasis
GHR	Growth Hormone Receptor	ENSG00000112964	Protein coding	A transmembrane receptor for growth hormone

Red color indicates positive correlation; blue color indicates negative correlation.
